# Somatic Genomic Mosaicism in Multiple Myeloma

**DOI:** 10.3389/fgene.2020.00388

**Published:** 2020-04-22

**Authors:** Christine J. Ye, Jason Chen, Guo Liu, Henry H. Heng

**Affiliations:** ^1^The Division of Hematology/Oncology, Department of Internal Medicine, University of Michigan, Ann Arbor, MI, United States; ^2^Center for Molecular Medicine and Genomics, Wayne State University School of Medicine, Detroit, MI, United States; ^3^Department of Pathology, Wayne State University School of Medicine, Detroit, MI, United States

**Keywords:** cellular heterogeneity, fuzzy inheritance, genome chaos, genome theory, macro-cellular evolution, two phases of cancer evolution, karyotype coding, system inheritance

## Background

Somatic genomic mosaicism occurs when somatic cells of the body display different genotypes (Table 1), it has recently received increased attention because of its implications in disease, including neurodegenerative diseases and Down syndrome (Iourov et al., 2008, 2010, 2019; Biesecker and Spinner, 2013; Hultén et al., 2013; Vijg, 2014; Campbell et al., 2015; Fernández et al., 2016). Genomic mosaicism also contributes to high levels of cellular heterogeneity in pathological conditions, which is a distinguishing feature of cancer (Heng, 2015, 2019). In fact, high heterogeneity in cancer represents an extreme example of genomic mosaicism.

**Table 1:** Definitions/terminologies.

**Genome theory** The Genome Theory is a genomic theory of inheritance. The main concept is that traits are passed from parents to offspring through genome package transmission. This departs from the gene theory where genes, representing independent informational units, determine the individual's characteristics. The Genome Theory considers genomic topology as the context for gene interactions, and genomic inheritance defines genomic network structure through karyotype coding. Importantly, under stress, the genomic topology can be altered by re-organizing the genome, leading to the emergence of new systems. Such mechanism is responsible for macroevolution both in somatic cell and organismal evolution (Heng, 2015, 2019; Shapiro, 2017).
**Somatic mosaicism vs. genomic heterogeneity** These two terms can refer to the same phenomenon when there are distinctive genetic or genomic cell populations within an organism. Traditionally, mosaicism is thought to occur during early development (both mitotically and meiotically). With increased observations of a high degree of mosaicism in adult tissues associated with normal and disease conditions, and the realization that mosaicism is a stress response needed for somatic evolution at all stages, the usage of somatic mosaicism starts overlapping with genetic/genomic heterogeneity. Here, “somatic mosaicism” rather than “genomic heterogeneity” is used to promote the exchangeable use of these two terms in cancer research.
**Karyotype coding vs. gene coding** Karyotype coding is responsible for passing system inheritance, while gene coding determines parts inheritance (Ye et al., 2019b). System inheritance is inherited by the order of genes/DNA sequences along/among chromosomes. In contrast, parts inheritance is stored by the order of base pairs within genes. System inheritance is species-specific, but parts inheritance can be shared among different species. The function of sexual reproduction preserves the karyotype coding through meiosis by checking the order of genes along paired chromosomes (Gorelick and Heng, 2011). In many diseases, somatic mosaicism at the karyotype level is common, suggesting the importance of altered genomic information in cellular populations. However, they have often been ignored due to the popularity of gene-centric concepts. Changing the karyotype coding is a hallmark of somatic and organismal macroevolution (Heng, 2019; Ye et al., 2019a).
**Macrocellular evolution vs. microcellular evolution** Macrocellular evolution refers to the punctuated cellular evolution often mediated by karyotype changes, while microcellular evolution refers to the stepwise cellular evolution mediated by gene mutations and epigenetic variations. The two phases of cancer evolution were initially documented by experiments of karyotype evolution in action and then confirmed by cancer genome sequencing (Heng et al., 2006; Heng, 2015). Note that studying punctuated clonal evolution should focus on karyotype profiles as karyotype change-mediated macroevolution differs from gene-mediated microevolution. The relationship between macro- and microevolution also illustrates the interactions among individual molecular mechanisms, genome heterogeneity, system stresses, and evolutionary phase transitions. For example, extremely high stress can change the evolutionary phase. Evolutionary tipping points are often detected within the stress-induced crisis stage, leading to phase transition events such as transformation, metastasis, or drug resistance. Immediately following the event of transition, the degree of heterogeneity falls to the lowest level, after which the growth of a more homogenous population dominates (Ye et al., 2018). The two-phased cancer evolution pattern also challenges the general assumption that the accumulation of microevolution over time leads to macroevolution (Heng, 2015, 2019).
**Genome chaos vs. chromothripsis** Genome chaos or karyotype chaos refers to a phenomenon of rapid and massive genome re-organization. Initially described in karyotype studies by watching evolution in action (Heng et al., 2006), this mechanism was confirmed by cancer genome sequencing, albeit mainly illustrated by identifying gene mutations or copy number variations. Many names have been introduced to describe these genome re-organization events, including “chromothripsis,” which is a subtype of genome chaos (Heng, 2019). High levels of stress during crises can trigger genome chaos, and the rapid and massive genome re-organization can lead to new survivable genomes essential for macroevolution. Overall, stress response-induced emergent systems and their adaptation is a key component of somatic cell evolution, which provides a unifying framework for understanding diverse molecular mechanisms.
**Adaptive systems** Complex systems, which are integrated by a set of interacting or interdependent parts or entities. Such whole systems are able to respond to environmental changes or changes in its own interacting parts (including the parts' topology), often in a non-linear fashion. The key features of adaptive biosystems include feedback loops, part heterogeneity, dynamic emergence, multiple levels of fuzzy inheritance, evolutionary capability, and uncertainty between part alteration and whole system behavior. Biological systems are typical adaptive systems which are much more difficult to predict than non-biological systems. The understanding of lower level parts usually does not lead to the understanding of a whole bio-system, especially its emergent behavior under crises (Heng, 2015, 2019).

The genomic basis of somatic genomic mosaicism, however, remains to be elucidated. Traditional explanations have focused on defective cellular processes, including imperfect DNA replication and repair, abnormal chromosomal machinery, and a faulty stress response to environmental challenges. As illustrated by the evolutionary mechanism of cancer (Ye et al., 2009), nearly all molecular pathways/mechanisms can contribute to variations in cellular systems. The conventional wisdom is that biosystems are not perfect and that error-generating opportunities exist. Thus, the major goals of molecular medicine have been to detect and fix these errors.

Nevertheless, bioerrors (or imperfect-biosystems) do not explain the high degree of genomic mosaicism revealed by large-scale -omics technologies (Vattathil and Scheet, 2016), and plausible mechanisms are not yet revealed (Heng et al., 2016). These novel mechanisms should address (a) both the positive and negative contributions of cellular heterogeneity in normal and disease conditions and (b) the survival strategy of cancer cells to drastically elevate the level of heterogeneity in crisis conditions. Using multiple myeloma (MM) as an example, these mechanisms will be examined in the context of bio-information, adaptive systems (Table 1), and emergent behavior during cancer evolution.

## A High Degree of Somatic Genomic Mosaicism, A Necessary and Sufficient Condition for Evolution, is Common in MM

MM patients display a high level of karyotype heterogeneity. Different patient genotypes can involve poly-aneuploidy, hyperdiploidy, hypodiploidy, chromosomal translocation, chaotic genomes (such as chromothripsis) (Table 1), and/or a combination of other gene mutations and chromosomal aberrations (Garcia-Sanz et al., 1995; Avet-Loiseau et al., 2007; Klein et al., 2011; Magrangeas et al., 2011; Keats et al., 2012; Bolli et al., 2014; Lee et al., 2017; Kaur et al., 2018; Smetana et al., 2018; Ashby et al., 2019; Maura et al., 2019).

Four key realizations from the Genome Theory (Table 1) can explain why such karyotype heterogeneity is observed in MM patients:

(1) Karyotype changes lead to new genomic information packages. According to the Genome Theory, the karyotype codes “system inheritance” (the genomic blueprint), while the genes code for “parts inheritance” (Table 1) (Ye et al., 2019b). Specifically, karyotype coding ensures the order of genes and other DNA sequences along and among chromosomes for a given species.
Karyotype coding changes can replace the function of a specific gene (Rancati et al., 2008) and impact global gene interaction, leading to new genome systems (Stevens et al., 2013, 2014). In MM, unique gene expression patterns are associated with recurrent chromosomal translocation and ploidy (Zhou et al., 2009). A recent cancer genome analysis has illustrated that the profile of chromosome aberrations is much more useful than gene mutation profiles when correlated with clinical outcomes either as prognostic or predictive markers (Davoli et al., 2017; Jamal-Hanjani et al., 2017). This result was also confirmed in MM, as karyotypic events have a stronger impact on prognosis than mutations (Bolli et al., 2018). In fact, chromosomal profiles have extensively been associated with prognosis in MM, based on specific translocation, hyperdiploidy, chromosomal amplification/deletion, and chromosomal copy number abnormalities (Garcia-Sanz et al., 1995; Avet-Loiseau et al., 2007, 2009; Walker et al., 2010; Shah et al., 2018). By converting DNA sequence data into aneuploidy data, we showed that the status of aneuploidy can suggest clinical MM outcomes (Ye et al., 2019a).
(2) Cancer often represents an evolutionary trade-off of cellular variation-mediated function. Since genomic variations are needed for cellular adaptation, and many essential bioprocesses often can generate harmful byproducts, genomic variations seem unavoidable. For example, normal B-cell development (affinity maturation in the germinal center) and antibody generation require somatic hypermutation and class-switch recombination. However, these key processes also generate DNA breaks and chromosomal translocations, which are central characteristics of MM (Manier et al., 2017). This represents an immune system trade-off: performing immune functions comes with the risk of malignant transformation [via translocation of cancer genes into immunoglobulin (Ig) loci and/or new karyotype formation] (Gonzalez et al., 2007).
(3) Even though heterogeneity has growth disadvantages (including in cancer), being highly heterogeneous is the winning strategy for most cancers. Genome chaos is essential for population survival under crises, even though it is extremely expensive due to the massive death and often slow growth of the cell population. The key is to create new survivable genomes (through macro-cellular-evolution) (Table 1), after which relatively homogenous growth will soon follow with the help of oncogenes in a stochastic fashion (through micro-cellular-evolution) (Ye et al., 2018; Heng, 2019). This principle is used to develop an MM model by synthesizing new patterns of clonal evolution as well as sequencing data (Manier et al., 2017; Maura et al., 2019; Ye et al., 2019d).
(4) The only way for a new system to emerge is to break the constraints above that system (e.g., cellular competition, tissue organization, immuno-systems, and chemo-drugs). In general, different genome systems are required to break different types of constraints (e.g., different karyotypes are involved during different stages of cancer evolution). It is also difficult for any new genome to become dominant. This high level of aberrated genomes therefore become a sufficient condition for cancer evolution.

In addition to the karyotypic level of mosaicism discussed, different types of somatic mosaicism include copy number variations (CNVs) (Walker et al., 2010, 2015; Lohr et al., 2014; Bolli et al., 2018; Aktas Samur et al., 2019), gene mutations (both driver and passenger) (Chapman et al., 2011; Egan et al., 2012; Keats et al., 2012; Bolli et al., 2014, 2018; Lohr et al., 2014; Walker et al., 2015), and non-genetic variations (e.g., epigenetic variations) (Huang, 2009; Heng, 2019). Together, the multiple levels of genetic variation represent the high degree of somatic genomic mosaicism in MM.

## The Main Mechanism of Somatic Genomic Mosaicism is “Fuzzy Inheritance” Which is Coded by Living Systems to Adapt to Microenvironmental Dynamics

Cellular heterogeneity has biological significance and genomic basis. Essential cellular heterogeneity is ensured by fuzzy inheritance, a key component of the self-regulating features in bio-adaptive systems. Specifically, heterogeneity is encoded by the genome and realized by genotype-environment interaction (even though bio-errors can also contribute).

Under classical inheritance theory, the gene codes for a fixed or defined genotype, while the environment can influence the real phenotype. For complex polygenic traits, many individuals are needed to illustrate the mode of inheritance. Unfortunately, as shown by the effort of the genome-wide association studies, the multiple genes that contribute to a polygenic trait are hard to identify despite huge sample sizes used. Many loci are involved, and each only contributes to a tiny portion of the phenotype.

To solve this confusion, the new concept of fuzzy inheritance was proposed: genes and chromosomes code for a potential range or spectrum of phenotypes, and the environment serves as a selective “scanner” to “choose” a specific phenotype among the many defined by the genotype (Heng, 2015, 2019). Although the environment plays an important role in phenotypic selection, it is limited by the range established by the inherited genotype: the ultimate phenotype can only be selected from that range. Since diseases are variable phenotypes defined by the interaction between genomic information and environment (Heng et al., 2016), a normal gene can produce a disease phenotype, and disease-associated gene mutations can display a normal phenotype, depending on the environment.

Interestingly, fuzzy inheritance and dynamic environmental interaction will likely be responsible for the majority of phenotypic plasticity. Given the importance of the microenvironment in MM, the role of fuzzy inheritance in cancer evolution should be a top research priority.

## The Importance of Somatic Genomic Mosaicism for New Emergent Genomes

Cellular heterogeneity can alter emergent properties, and cells that diverge from the average population—outliers—often define the direction of cancer evolution (Heng, 2015, 2019). However, cancer researchers have traditionally ignored the contribution of outliers and focused solely on average profiles or dominant clones. Under normal developmental or physiological conditions, this approach may work (although one must note that, even under normal conditions, the 80/20 principle where about 80% of the effects come from 20% of the causes can still play a role). However, under pathological conditions, especially under cellular crisis conditions, some outliers, such as cells with extremely different phenotypes, often become the dominant population. The general conditions for tipping the balance include new altered genomes that favor survival, environmental constraint, and status of the mosaicism. Interestingly, under the right conditions, even a slight change can trigger the tipping point. For example, when the proportion of outliers in the cellular population changes, even in the range of a few percent, an evolutionary phase transition can occur. Such tipping-point system behavior significantly increases the success of cancer evolution when high heterogeneity exists in the cellular population (Maura et al., 2019). When combined with the difference in initial conditions, cellular heterogeneity makes it very hard to predict the outcomes for most cancer cases.

Equally important, since different subpopulations can be molecularly profiled, especially after becoming dominant clones, a huge number of molecular mechanisms can be characterized. Data from recent studies illustrate diverse genetic variations in MM disease evolution (Egan et al., 2012; Keats et al., 2012; Bolli et al., 2014, 2018; Pawlyn and Morgan, 2017; Aktas Samur et al., 2019; Maura et al., 2019). A better way to understand MM is to study the evolutionary mechanism of cancer (Ye et al., 2009), rather than continue identifying individual molecular mechanisms: when there are so many, the clinical prediction of any single mechanism is low due to highly dynamic evolutionary processes.

## The Clinical Implications of Genomic Somatic Mosaicism and System Constraint

First, it is important to identify the phase of evolution before initiating or changing treatment. Since different types of inheritance are directly related to micro- and macro-somatic evolution, and all cancer phase transitions are defined by macrocellular evolution, the selection of new systems is significantly different from selection on individual genes, especially since the function of any individual gene is influenced by its genomic context. The relationship between disease progression (from MGUS, smoldering MM to active MM) and evolutionary pattern (micro-and macro-somatic evolution) of MM remains to be determined. This will guide when and how to intervene at different stages of the disease in different subpopulations of patients (Table 1).

Applying somatic mosaicism in the clinic represents a new approach. On the surface, it is challenging to directly target mosaicism compared to a molecular pathway. However, this seeming disadvantage is actually an advantage when dealing with adaptive systems in which many pathways are involved (e.g., when the causative role for any pathogenic effect is difficult to elucidate and therapies can lead to toxicity and/or secondary malignancies).

In the case of MM: it is worthwhile to investigate whether asymptomatic patients at the stage of smoldering MM can be distinguished by mosaicism. Of course, it is also possible that this clinical challenge will remain even after analyzing evolutionary profiles. Only future investigations will tell.

Second, the stability of higher systems above cancer cells, i.e., the broader microenvironment, organ system, and immune system, can be applied to constrain cancer evolution by slowing down or stabilizing the specific phase of evolution. As all medical treatment can function as cellular stress that may alter the system's evolutionary dynamics (Kultz, 2005; Horne et al., 2014), caution is crucial when weighing the impact of treatment in the context of evolution. For example, within the stable micro-evolutionary phase, moderately treating cells is a better approach than maximal killing, as an over-killing strategy will trigger genome chaos, leading to rapid drug resistance (Heng, 2015, 2019). MM resistance is frequently associated with chromothripsis (Lee et al., 2017) and likely involves treatment-induced genome chaos. Thus, therapies using an adaptive strategy might confer better long-term benefits (Gatenby et al., 2009; Lohr et al., 2014). So far, clinical trials using adaptive strategies in MM treatment (moderate dosage and treatment schedule) have been explored and likely to yield better clinical outcomes (Ye et al., 2019c). On the other hand, instead of putting stress or therapeutic pressure directly on cancer cells, using immunotherapy to modulate the cancer microenvironment (to enhance immune cytotoxic effects and system constraint) is an attractive strategy.

## Author Contributions

CY and HH drafted the manuscript. JC and GL participated in the discussion, literature search, and editing of the manuscript.

## Conflict of Interest

The authors declare that the research was conducted in the absence of any commercial or financial relationships that could be construed as a potential conflict of interest.
